# Standardising the reporting of outcomes in gastric cancer surgery trials: protocol for the development of a core outcome set and accompanying outcome measurement instrument set (the GASTROS study)

**DOI:** 10.1186/s13063-017-2100-7

**Published:** 2017-08-09

**Authors:** Bilal Alkhaffaf, Anne-Marie Glenny, Jane M. Blazeby, Paula Williamson, Iain A. Bruce

**Affiliations:** 1 Department of Oesophago-Gastric Surgery, Manchester Royal Infirmary, Central Manchester University Hospitals NHS Foundation Trust, Manchester Academic Health Science Centre, Manchester, UK; 2 Division of Molecular and Clinical Cancer Sciences, University of Manchester, Manchester, UK; 3 Division of Dentistry, University of Manchester, Manchester, UK; 4 Centre for Surgical Research, School of Social and Community Medicine, University of Bristol, Bristol, UK; 5 MRC North West Hub for Trials Methodology Research, University of Liverpool, Liverpool, UK; 6 Paediatric ENT Department, Royal Manchester Children’s Hospital, Central Manchester University Hospitals NHS Foundation Trust, Manchester Academic Health Science Centre, Manchester, UK; 7 Division of Infection, Immunity and Respiratory Medicine, University of Manchester, Manchester, UK

**Keywords:** Stomach neoplasms, Surgical oncology, Patient outcome assessment, Qualitative research, Consensus, Outcome assessment (Health care)

## Abstract

**Background:**

Gastric cancer is one of the leading causes of cancer-related deaths worldwide. Whilst surgery is the mainstay of curative treatment, it is associated with significant risks. Surgical strategies for treating gastric cancer should be based on evidence from systematic reviews of well-designed randomised controlled trials. However, inconsistencies in the reporting of outcomes from these trials makes evidence synthesis unreliable. We present a protocol for an international consensus study to develop a standardised set of outcomes and measurement tools – a ‘core outcome set’ (COS) – to be used by all future trials examining therapeutic surgical interventions for gastric cancer. The GASTROS study aims to standardise the reporting of outcomes in gastric cancer surgery trials through an international consensus process of key stakeholders including health care professionals and patients.

**Methods:**

The first of three stages in the study will identify a ‘long-list’ of potentially important outcomes to be prioritised. These will be extracted from a systematic review of relevant academic literature and patient interviews. Stage 2 will comprise an eDelphi survey which will consider the views of patients, nurse specialists and surgeons to prioritise the most important outcomes. A meeting of stakeholder representatives will ratify the COS.

Stage 3 will focus on identifying appropriate instruments to measure the prioritised outcomes by means of quality assessment of available measurement instruments and stakeholder consultation.

**Discussion:**

This study aims to standardise the reporting of outcomes in future trials examining therapeutic surgical interventions for gastric cancer. It is anticipated that standardisation of outcome reporting in these surgical effectiveness trials will enhance the evidence base for clinical practice. Highlighting outcomes of greatest importance to patients will ensure that their perspectives are central to research in this field.

**Electronic supplementary material:**

The online version of this article (doi:10.1186/s13063-017-2100-7) contains supplementary material, which is available to authorized users.

## Background

Gastric cancer is one of the leading causes of cancer-related deaths worldwide [1] and despite developments in multimodal treatment approaches, overall survival rates have not improved significantly over the last four decades [2]. Surgery to remove part or all the stomach continues to be the mainstay of treatment that offers a potential cure; however, this is associated with significant risks of short and long-term complications [3, 4]. Variations in surgical approaches aim to minimise these risks without compromising the oncological resection of the tumour. These variations can be broadly categorised into those related to accessing the stomach (e.g. open, laparoscopic or robotic surgery) and those related to the extent of surgery (e.g. partial or total gastrectomy, level of lymphadenectomy and whether splenectomy is r﻿equired).

In principle, assessing the optimal surgical strategies for gastric cancer should involve analyses of well-designed and well-conducted randomised controlled trials (RCTs) with systematic reviews and meta-analyses of data. However, trials are often methodologically heterogenous, report and measure their outcomes differently and preclude comprehensive evidence synthesis. Consequently, strong recommendations for clinical practice can seldom be made [5, 6]. In instances where trials may report the same outcomes, the definitions of these outcomes are often inconsistent and it is not known to what degree these outcomes may be relevant to key stakeholders such as patients.

In preparing this protocol, a rapid review of RCTs (published between January 2014 and January 2016) examining therapeutic surgical interventions for gastric cancer was undertaken using a structured search strategy applied to MEDLINE and EMBASE via Ovid. In the six trials identified, a total of 102 outcomes were reported, only 15% of which were defined. No single outcome was reported by every trial and only one trial described patient-reported outcomes. No trial measured quality of life after surgery.

Many groups have now demonstrated similar, widespread inconsistencies in outcome reporting [7–10]. Consequently, there has been a drive, with the support of initiatives such as COMET (Core Outcomes Measurement in Effectiveness Trials) and the Medical Research Council’s Hubs for Trials Methodology Research, to standardise the reporting of outcomes as an important step in improving trial design and reducing research waste.

### Aims and objectives

One solution to this problem is through the development of a ‘core outcome set’ (COS). A COS is defined as an agreed *minimum* set of outcomes that should be measured and reported in all trials in a specific condition [11]. The aim of the GASTROS study (GAstric cancer Surgery Trials Reported Outcomes Standardisation) is to develop a COS to be used by all trials examining therapeutic surgical interventions for gastric cancer, which reflects the interests of both patients and health care professionals.

The specific objectives include:

1. To determine the degree of variation in the reporting of outcomes in the academic literature
2. To identify a list of potentially important outcomes from published trials and trial protocols
3. To identify a list of potentially important outcomes reported by patients, in semi-structured interviews, who have been treated for gastric cancer, to augment the list generated in item 2
4. To reach consensus regarding the most important outcomes from the perspective of patients and health care professionals into a COS
5. To identify appropriate outcome measurement instruments (OMIs) to be used in the reporting of the COS and at what time points the outcomes should be measured

## Methods

This study will draw its methodological principles from recommendations developed by initiatives such as COMET and COSMIN (COnsensus-based Standards for the selection of health Measurement INstruments) and modified where necessary and appropriate [12–17]. The COMET initiative has been instrumental in propagating the agenda for change in relation to outcomes reporting internationally and has amassed a wealth of knowledge and experience during the last 6 years. Whilst the field of COSs is still relatively new, COMET is supporting the development of hundreds of COSs demonstrated by over 400 completed, ongoing or planned studies across a wide spectrum of clinical specialties referenced in its online database [18]. COSMIN, whose focus lies on developing rigorous methods of OMI selection, is working closely with COMET and has developed standards for selecting instruments used in the reporting of COSs [16].

### Scope

This COS is primarily aimed at pragmatic trials examining therapeutic *surgical* interventions for gastric cancer. The target population is male and female adults. We foresee that the COS will also be beneficial for the design of non-randomised studies and will inform the design of databases and national audits by identifying the priorities of patients and health care professionals.

Given that there is now a greater acceptance that the management of gastric cancer is often multimodal (involving a combination of surgical excision and chemotherapy or chemo-radiotherapy) [19], it may be argued that a COS would be more relevant if it were to encompass *all* therapeutic interventions and not just *surgical* ones. A structured search of the World Health Organisation’s International Clinical Trials Registry Portal (http://apps.who.int/trialsearch/, last accessed 3 August 2016) and ClinicalTrials.Gov (https://clinicaltrials.gov/, last accessed 3 August 2016) has identified 24 ongoing *surgical* gastric cancer trials planning to recruit approximately 11,000 patients. The rate at which these surgical trials are being set up does not show signs of slowing. As such, a surgically focused COS is highly relevant given the research activity within this field. Furthermore, there is a significant proportion of patients who do not require multimodal therapy due to early stage disease or on the account that they are unfit for additional therapies. In addition, given the large *variation* in surgical practice that already exists, and the range of therapeutic surgical interventions which have been, and are being, investigated, we believe that a surgically orientated COS is both desirable and necessary. Nonetheless, our group recognises that future work is required to develop a COS which will be relevant to non-surgical interventions and this is within our planned programme of work in conjunction with endoscopic and medical and clinical oncology groups.

### Definitions

The development of COSs by different groups has highlighted some of the issues which arise with the inconsistent use of nomenclature and definitions in a new and developing research field. There are no widely agreed definitions for several commonly used terms in COSs; however, the COMET initiative recommends that studies clearly define their own terms. Our definitions are summarised in Table 1.

**Table 1:** Definition of terms used in the GAstric cancer Surgery Trials Reported Outcomes Standardisation (GASTROS) study

Core outcome set (COS)	An agreed *minimum* set of outcomes that should be measured and reported in all trials in a specific condition [11]
Outcome	A unique endpoint which attempts to describe health-related changes that occur secondary to a therapeutic intervention, e.g. hospital-acquired pneumonia
Outcome domain	A collection of ‘outcomes’ which share common features, e.g. the outcome domain ‘respiratory complications’ would include outcomes such as ‘pleural effusion’, ‘hospital-acquired pneumonia’ and ‘atelectasis’
Outcome measurement instrument (OMI)	A method or tool used to measure an ‘outcome’ or an ‘outcome domain’
Outcome measurement instrument set	A collection of OMIs which are used to measure outcome domains in a COS

### Stakeholder involvement

An important aspect of the GASTROS study design is ensuring that key stakeholder opinion is represented at every stage of the COS development. Our primary stakeholder groups include patients (with ‘lived experience’ of the condition and its management), surgeons (those directly delivering and developing the clinical interventions) and clinical nurse specialists (as they have an important dual role as health care professionals and patient advocates). In addition to their participation highlighted below (see ‘Study design’), representatives from each group have been recruited to our Study Advisory Group to support the general delivery of the study against its stated objectives and ensure that the viewpoints of all stakeholders are considered throughout the process.

Most surgical gastric cancer trials are undertaken in the Far East where the incidence and prevalence is highest [1]. It is, therefore, essential that aspects of the COS development take this international perspective into account. As such, representatives from all stakeholder groups will be invited to participate in the eDelphi survey. They will be drawn from a broad network of national and international patient groups, charities, professional associations and institutions. This is further elaborated upon below (see ‘Dissemination and implementation strategy’).

### Study design

**Fig. 1:**
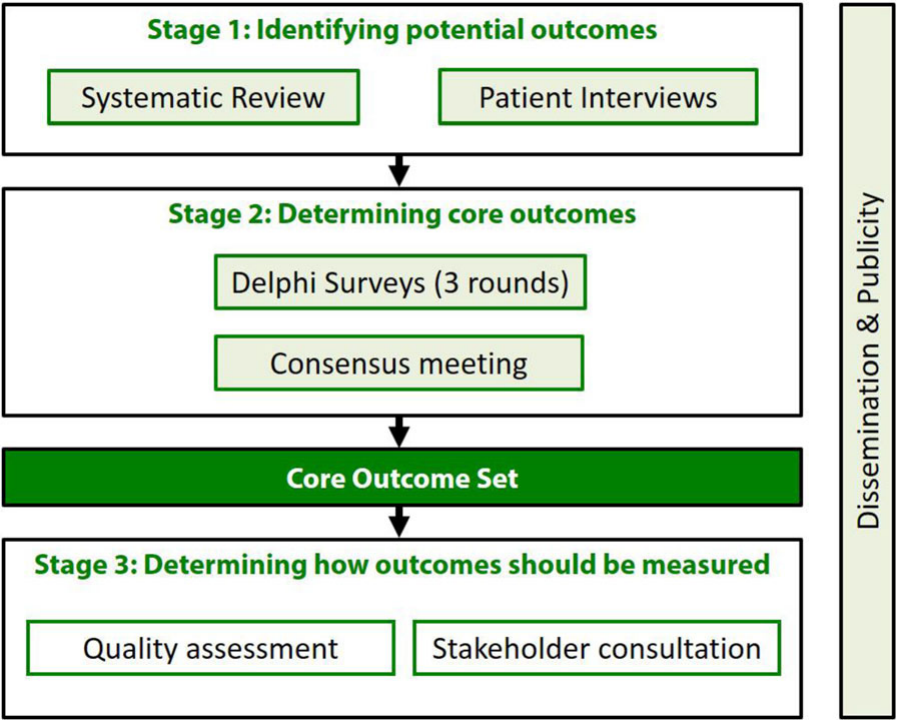
GAstric cancer Surgery Trials Reported Outcomes Standardisation (GASTROS) study overview

The GASTROS study will be divided into three distinct stages, summarised in Fig. 1:

- Stage 1. Generation of ‘long-list’ of outcomes
- Stage 2. Prioritisation of outcomes and finalisation of a COS
- Stage 3. Identification of OMIs

### Stage 1. Generation of ‘long-list’ of outcomes

A long-list of potentially important outcomes will be identified by means of a systematic academic literature search and semi-structured patient interviews. This will be followed by a consultation exercise to finalise a list of outcomes to be prioritised by stakeholders in stage 2 of the study.

### Systematic academic literature search

A systematic review of randomised control trials (RCTs) and protocols of RCTs examining therapeutic surgical interventions for gastric cancer will be undertaken. Systematic reviews of RCTs will be scrutinised to identify publications not previously identified. We will limit our analysis to RCTs as the primary purpose of the GASTROS study is to influence future RCTs.

Abstracts will be screened by two researchers and relevant publications identified. All reported outcomes will be extracted verbatim in addition to definitions and OMIs used. Validated Patient-reported Outcome Measurements (PROMs), such as those used to report ‘quality of life’, will be critically reviewed to identify further outcomes to be added to the ‘long-list’ [20]. Whilst overall quality of life may be deemed an outcome that should be prioritised and included in a COS, there may be components related to eating and drinking, for example, that may be deemed important outcomes within their own right.

### Patient interviews

Previous reports have highlighted that patients often have differing priorities and perspectives relating to outcomes measured in trials [21–24]. To ensure that the views of patients are adequately considered, a series of semi-structured qualitative interviews, with patients who have previously undergone surgery, will be undertaken. All interviews will be audio-recorded, transcribed and interrogated for themes which may supplement the outcomes already gathered from the systematic academic literature review. There is no ‘sample size’ calculation for qualitative research. The total number of participants should be guided by the concept of ‘saturation’, whereby further interviews do not result in the identification of new outcomes, and can range from between 5 and 50 participants [25, 26]. Based on the authors’ experience in qualitative research methods in COS development, we expect that between 15 and 30 patients will need to be interviewed before ‘saturation’ is reached.

To ensure that a broad range of views are expressed during the interviews, we aim to purposefully sample patients based on several characteristics. These include age, sex, time since surgery, type of surgical approach (open or minimally invasive) and whether patients have undergone other perioperative therapies (chemotherapy or chemo-radiotherapy).

### Stage 2: Prioritisation of outcomes to finalise a COS overview

Delphi surveys have been used in many COS projects to reach consensus on the most important outcomes to include [14, 27, 28]. One of the main benefits of this approach is that the views of all participants are equally heard. This may not be the case in a face-to-face forum where the views of one individual or group of participants may be more vociferously asserted. There is no fixed methodological approach to undertake a Delphi survey. Some groups have retained all potential outcome domains in each round and used the participant responses to inform a final consensus meeting [27, 29], whilst others have only retained outcome domains deemed important in each round [28]. We intend to use a hybrid approach over three rounds as described in greater detail below. Following the Delphi survey, a meeting of key stakeholder representatives will take place to ratify the prioritised outcomes into a COS.

### Organising the outcome list in preparation for stage 2

Once potentially important outcomes have been identified from the systematic review and patient interviews, a final long-list of items will be compiled for the eDelphi survey. We plan to recruit at least 100 participants and so to minimise non-response and attrition between survey rounds, the initial number of items submitted to the Delphi survey will need to be carefully managed. Previous COS developers have aimed for less than 100 initial items for participants to prioritise [28]. To achieve this, individual outcomes will be organised into ‘outcome domains’ (Table 1) whilst ensuring that domains do not become too broad. For example, the outcomes ‘hospital-acquired pneumonia’, ‘pleural effusion’ and ‘atelectasis’ grouped together under the outcome domain ‘respiratory complications’ may be appropriate, whereas grouping the same outcomes under the domain ‘complications’ may be too specific. The process of compiling and finalising the outcome domains will be undertaken during a meeting of key stakeholder representatives to ensure transparency. This meeting will involve open discussion of each outcome domain, including information relating to how the outcome domain was formulated, to ensure that it is not too broad or specific. An outcome domain will be admitted into the long-list for the subsequent eDelphi survey once agreement by majority regarding its appropriateness has been reached by all stakeholder representatives.

Each item entered into the survey will be described in lay terms with an additional scientific description. For example, an ‘anastomotic leak’ may be described as ‘a leak from the join between the stomach and the bowel’. All item descriptions will be reviewed by the study group and patient representatives. Items will be presented to participants as collections with similar characteristics (e.g. outcomes related to ‘adverse events’ or ‘technical aspects of surgery’).

### Participants and sample size

Representatives from our three primary stakeholder groups – patients, clinical nurse specialists and surgeons – will be invited to participate in the Delphi survey. Whilst there is no accepted or required ‘sample size’ requirement for a Delphi survey [30], we aim to recruit at least 100 participants in total. The views of each stakeholder group will be considered separately which will enable intra- and inter-stakeholder group variability to be explored. As explained previously, gastric cancer is a worldwide disease, and as such, participants will be sought internationally through a network of patient groups, organisations, professional associations and cancer institutes. The Delphi survey will be Internet-based; however, we will give participants the opportunity to complete hard copies of the surveys so as not to exclude those with limited Internet access or knowledge of the Internet.

### Delphi survey

#### Round 1

**Fig. 2:**
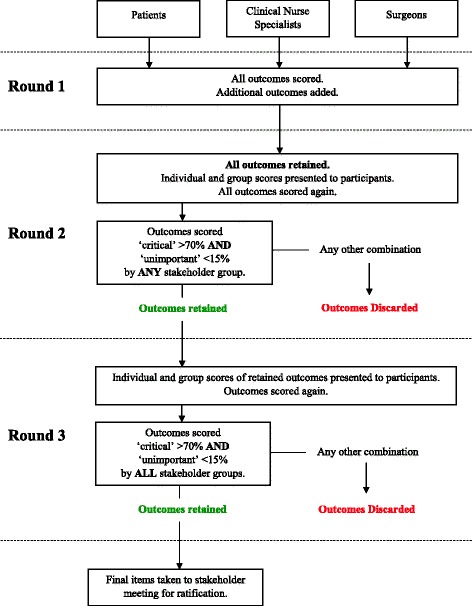
Summary of the Delphi survey process

A summary of the entire eDelphi survey process is illustrated in Fig. 2. Participants will be asked to score each outcome domain on a 9-point scale proposed by the GRADE group (http://www.gradeworkinggroup.org), in which 1 to 3 signifies an outcome of ‘limited importance’, 4 to 6 ‘important but not critical’, and 7 to 9 ‘critical’. Round 1 will also provide participants with the opportunity to add further outcomes which they think may be important. Any suggested outcomes deemed to represent a new outcome domain by the study group (following discussion and a majority decision) will be added to the list for consideration in round 2. In addition, prior to commencing round 1, participants will be asked to enter demographic information about themselves including country of residence and language spoken. Surgeons will also be asked about the volume of gastrectomies performed. This will enable us to explore the impact of language, cultural variation and surgical experience in relation to the Delphi survey responses.

#### Round 2

All items in addition to further new outcome domains identified by participants in round 1 will be carried forward for consideration in round 2. Descriptive statistics will be used to summarise the scores from round 1 and presented to participants. Participants will see the results of their individual score for each outcome in addition to the median score of each stakeholder group. The rationale for showing participants the scores from other groups is that it may improve consensus between the stakeholder groups [15]. In addition, by carrying all items forward from round 1, it may be possible to identify changes in scoring patterns as a result of viewing other scores. Participants will be asked to score all items once again using the 9-point scale.

Outcome domains which are scored ‘critical’ by greater than 70% of participants from *any* stakeholder group, *and* ‘unimportant’ by less than 15% of the group, will be carried forward for further consideration in round 3. The rationale for this threshold is that for an outcome domain to be included in the COS, it requires agreement by the majority regarding the critical importance of the outcome, with only a small minority considering it to have little importance. By carrying forward outcomes relevant to at least one stakeholder group, participants will be given another opportunity to reflect on the importance of the outcome domain in the final round. As the scope of this study is to identify the *most* important outcomes, all other outcomes will be discarded.

#### Round 3

All retained outcomes will be summarised and participants will view both their individual scores and those of the other groups before being asked to score items a final time using the 9-point scale. Outcome domains which are scored as ‘critical’ by greater than 70% *and* ‘unimportant’ by less than 15% of participants from *all three groups* will be retained for inclusion in the COS.

### Missing responses

If a participant does not complete a subsequent round of the Delphi survey, their scores from previous rounds will be counted as valid and retained in the study. Similarly, if a participant fails to score a specific item during a survey round, the answers to other items will be held as valid and retained. The rate of missing responses will be reported with the results of the Delphi survey.

### Stakeholder meeting

Following the Delphi survey, a meeting of stakeholder representatives will take place to review the results and recommend the outcome domains as a COS.

### Stage 3: Identification of outcome measurement tools

**Fig. 3:**
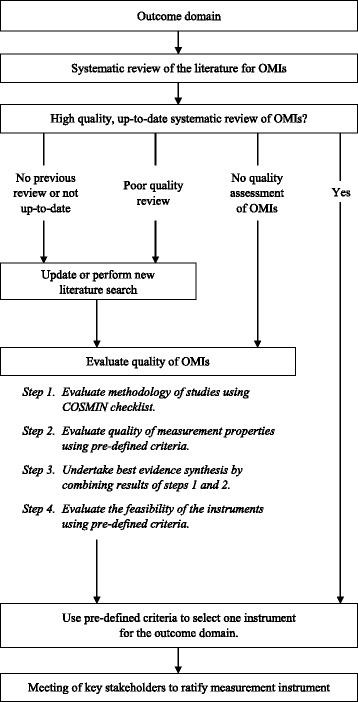
Process of identifying outcome measure instruments (OMIs) for outcome domains in the core outcome set (COS)

The final stage of the study will be based on guidance set out by COMET and COSMIN in the selection of appropriate measurement instruments for the outcome domains included in the COS [16]. Our strategy is summarised in Fig. 3 and is involves four stages;

1. Conceptual considerations
2. Finding existing OMIs
3. Quality assessment of OMIs
4. Recommendations on the selection of OMIs for a COS and at what time points they should be used

#### 1. Conceptual considerations

The first step involves identifying the scope of outcomes to be measured. These will be identified in stage 2 of the study. The scope of the COS has been described earlier in this protocol.

#### 2. Finding existing outcome measurement instruments (OMIs)

Existing OMIs will be identified through several approaches. A structured search of MEDLINE and EMBASE via Ovid will identify systematic reviews of OMIs for the outcome domain concerned. If the systematic reviews are of high quality and have undertaken a quality assessment of the OMIs, then one OMI will be selected and presented to a group of key stakeholder representatives at the end of the process.

If there are no systematic reviews or they are of poor quality, then a new or updated academic literature search will be performed. We will search MEDLINE and EMBASE via Ovid to identify studies of OMIs. We will also interrogate reference lists and examine trials and protocols identified in stage 1 of the GASTROS study to identify further OMIs. Studies describing these OMIs will be quality assessed as described below. OMIs identified through up-to-date systematic reviews where quality assessments were not undertaken will also be assessed by the predefined standards below.

#### 3. Quality assessment of OMIs

Each OMI-related study identified will undergo an evaluation of its methodological quality using the COSMIN Checklist [31]. In addition, an evaluation of the measurement properties of the OMI will be undertaken against several predefined criteria [16] including ‘content validity’ and ‘internal structure’. Content validity is defined as ‘the degree to which the content of a measurement instrument is an adequate reflection of the outcome to be measured’. ‘Internal structure’ is comprised of two aspects – ‘internal consistency’ (the degree of interrelatedness among the items within the OMI) and ‘structural validity’ (the degree to which the scores of a measurement instrument are an adequate reflection of the dimensionality of the outcome to be measured). If either ‘content validity’ or ‘internal structure’ are considered poor or unknown, then the OMI will not be assessed further. The results of this quality assessment will be combined in a ‘best evidence synthesis’ exercise against criteria defined by the COMET-COSMIN guidance.

It is also essential that OMIs are assessed in terms of their feasibility of use. The COMET-COSMIN guidance provides 17 different factors against which feasibility can be assessed. These include ‘patient comprehensibility’, ‘interpretability’, ‘ease of administration’ and ‘completion time’.

#### 4. Generic recommendations on the selection of outcome measurement instruments for a COS

An OMI will be recommended if it meets the following criteria:

1. There is ‘high-quality’ evidence of ‘good’ content validity and ‘good’ internal structure
*and*
2. The OMI is feasible to use

‘High-quality evidence’ is defined as consistent findings in multiple studies of at least ‘good’ quality *or* in one study of ‘excellent’ quality *and* a total sample size of 100 patients or more (see the COSMIN Checklist [31] for clarification of the terms ‘good’ and ‘excellent’ quality).

It is possible that more than one OMI can be recommended for an outcome domain. Conversely, it is possible that no OMIs are recommended. This scenario may form the basis of future work to develop an OMI for that domain.

#### Key stakeholder meeting

Following quality assessment for OMIs for each outcome domain included in the COS, we will invite representatives from each key stakeholder group to review the evidence from this stage of study and ratify the recommended OMIs as an OMI set. The primary function of the stakeholder meeting is to ensure transparency of the process, raise further questions and seek further clarifications (if any). The evidence considered will also inform recommendations made through the stakeholder meeting regarding when these OMIs should be used to measure the core outcomes.

### Implementation strategy

A COS must be implemented widely within its clinical field to have its intended benefit. Whilst grant-awarding bodies and international research groups are increasingly promoting the use of COSs, researchers must be willing to incorporate them into trial designs. Our approach to maximise the use of our COS is one of inclusion of key stakeholders in designing and delivering our study and dissemination of the findings at every stage. Given that most surgical gastric cancer trials are being undertaken in the Far East, this inevitably means involvement of international stakeholders. We are working with several groups in South Korea, Japan and China in addition to European and North and South American teams to ensure that this aspect of our study is facilitated. It is not yet fully understood how language or cultural differences may affect the results of consensus processes such as the one we propose. Our study will provide the opportunity to explore this question further.

Some of the steps that we have considered as part of our dissemination and implementation strategy include:

1. Registration of our study with COMET database (http://www.comet-initiative.org/studies/details/764?result=true)
2. Development of our study website (www.GASTROSstudy.org) where key stakeholders and interested parties can find regular updates and register for participation
3. Development of our social media identity, e.g. Twitter (@GASTROSstudy)
4. Widespread dissemination of our work at every stage of the study through:
  - National and international scientific meetings
  - Journal publications
  - Patient events
  - Regular updates to our network of international patient groups and charities, professional associations and cancer centres

Engagement with, and ‘ownership’ of, the COS by professional bodies will also be an important way to facilitate the necessary regular review of the COS. Such reviews are needed to ensure that individual outcomes remain relevant and to add new outcomes as appropriate. No recommendation exists regarding the time interval between reviews, but we anticipate the need for review within 3–5 years.

## Discussion

There is no COS for trials examining surgical interventions for gastric cancer. Through the GASTROS study, we aim to standardise the definition, collection and measurement of core outcomes which can be used to compare future trials in this field. This will:

1. Improve the reliability of evidence synthesis on which robust clinical guidelines can be based
2. Improve shared decision-making and the preoperative consent process as outcomes from surgical interventions which are relevant to both clinicians and patients become more apparent
3. Better equip health care providers how best to prioritise funding for interventions that reflect the needs and priorities of patients

The COS will also inform non-RCT trial design and, additionally, provide a minimum set of outcomes relevant to key stakeholders which can be collected by health care providers and organisations designing national audits and prospective databases.

The GASTROS study will also provide a platform for future work which includes the development of PROMs where they are deficient and the further development of a COS which is relevant to multimodal therapies. Once core outcomes are identified, work can also commence on developing a minimum dataset of factors which can influence these outcomes so that risk adjustment of outcomes and ultimately the external validity of trials can improve.

## Additional file

Additional file 1: SPIRIT checklist for GASTROS study protocol (Additional file 1). (DOCX 52 kb)
